# An investigated organic and inorganic reinforcement as an effective economical filler of poly (methyl methacrylate) nanocomposites

**DOI:** 10.1038/s41598-022-20393-3

**Published:** 2022-09-30

**Authors:** Heba I. Elkhouly, Eman M. Ali, M. N. El-Sheikh, A. El-Sayed M. Hassan

**Affiliations:** 1 Department of Mechanical Engineering, Faculty of Engineering, Beni-Suef University, Beni-Suef, Egypt; 2 Production Technology Department, Faculty of Technology and Education, Beni-Suef University, Beni-Suef, Egypt

**Keywords:** Mechanical engineering, Materials science

## Abstract

Polymer matrix composites have garnered the interest of the dentistry sector. Nano-fillers are frequently used as reinforcements in these composites to enhance their characteristics. Poly (methyl methacrylate) was filled with date seed nanoparticles (DSNP) and titanium oxide nanoparticles (TiO_2_NP). In this work, two nanofillers (DSNP and TiO_2_NP) were analyzed using Fourier-transform infrared spectroscopy (FTIR). In addition, the features of the PMMA-nanofiller composite were experimentally evaluated via compression, micro-hardness, wear rate (WR), and coefficients of friction (µ) testing. Utilizing a scanning electron microscope (SEM), the microstructure of the PMMA-DSNP composite was examined. The results of the experiments on the nanocomposites demonstrated that the elastic modulus, microhardness, wear resistance, and friction resistance increased with an increase in DSNP content to 1.2 wt, in comparison to TiO_2_NP at the same concentration. Finally, according to the guidelines, the ideal weight was determined to be 1.2 wt%, filler in the form of DSNP, at a normal load of 10 N.

## Introduction

Polymer matrix composites (PMC) have emerged as a major composite material, and particle-reinforced PMCs have attracted great interest owing to their exceptional technical features^1^. The polymer matrix possesses exceptional mechanical qualities, such as extraordinary tensile strength and self-healing capabilities. Moreover, a polymer matrix has more sensitive thermal characteristics than a metal matrix^2,3^. These composites are also popular in many engineering and medical applications due to their low cost and lightweight. By adding the best fillers to this matrix, the properties of these polymer materials can be improved, allowing the composite materials to be used in more place^4^.

PMMA has been utilized for decades as a polymer matrix material in composites. Due to its superior biocompatibility and simplicity of production, PMMA is widely recognized in the dentistry and medical industries. Additionally, it has poor mechanical qualities^5^. Inside, PMMA is the most often utilized material for removable denture bases. The PMMA polymeric base material is the most common of all dentures base materials because of its laboratory manipulation, polishing, and finishing capabilities, making it less expensive than other materials and providing oral stability and a fair look^6^. Alla and colleagues^7^ have become more common because they are made from a resin-based polymer system^8^.

In addition, dentists and patients are interested in plaque accumulation on removable acrylic equipment because of their food-absorbent structure and surface porosity, which promotes the bacterial activity of cariogenic oral flora^9,10^. Various treatments have been offered to improve the mechanical qualities of acrylic appliances and reduce the risk of dental cavities during orthodontic treatment. Orthodontic appliances made of antibacterial self-cleaning dental composite materials are one way that has recently gained popularity^11^. With this technology, many different fillers are added to orthodontic materials to improve their mechanical properties and antibacterial abilities^12^. In general, several fillers can be either inorganic or organic.

On the other hand, the most common inorganic fillers are, e.g., TiO_2_NP, SiO_2_NP, ZnONP, Al_2_O_3_NP, and halloysite nanotubes^13–16^. Furthermore, TiO_2_NP are non-toxic and chemically inert and have a high refractive index, corrosion resistance, hardness, and antibacterial activity. They can be synthesized in various configuration ^17–19^.

Moreover, Alrahlah et al.^20^ studied and evaluated a PMMA denture base material modified with TiO_2_NP in nano-mechanical, creep-recovery, and relaxation. As a result, TiO_2_ nanofiller also improved the antimicrobial properties of PMMA by significantly reducing bacterial adherence with an increasing TiO_2_ ratio.

While organic filler is produced from recycled waste materials such as nano seed powder of dates seed, pomegranate peels^21^, and fly ash^22^. These materials have been incorporated into various biomaterials to induce antimicrobial activity and improve mechanical behavior^23^. Moreover, organic fillers are becoming increasingly popular as environmentally friendly and renewable alternatives^24^.

The date seed fruit (Phoenix dactylifera L.) is one of the oldest plants cultivated, from the earliest records of Predynastic Egypt. The cultivation of date palms in Egypt goes back thousands of years^25^). The world makes about 9 million metric tons of dates yearly, but at least 2 million metric tons are wasted because there aren't many ways to use the seed^26,27^. The Middle East and North Africa are home to about 90 percent of the 100 million date palm trees grown for their fruit. Date seeds have a lot of mannan particles, which is a type of hemicellulose. But they haven't been studied as much as pomegranate peels and rice husk ash^28^, all common natural materials that can be used in many different ways. Among the many advantages of using fillers such as date seed nanoparticles (DSNP) are their low density, cost savings, and mechanical qualities throughout the manufacturing process (DSNP). Because of their intrinsic qualities, such as high chemical inertness and outstanding mechanical properties^20,29^, date seed particle reinforcements are considered attractive for manufacturing polymer matrix composites^30^. According to Elkhouly et al.^31^, the mechanical parameters of polymer composites augmented with DSNP fillers and two inorganic fillers were compared. According to the study results, this filler is competitive with inorganic fillers.

Zahedi et al.^32^ investigated two natural nanometer powders (almond shell flour) with different weight fraction ratios in a polypropylene polymer. The results showed that the values of these qualities improved for both groups of bio-composite specimens. Inside, Yuan et al.^33^ explained that poly (ethylene oxide) (PEO) was blended with PMMA and organically modified palygorskite, resulting in a composite polymer. The composite polymer membrane features a three-dimensional network structure^34^.

Literature reviews reveal that scientists explored the effects of various nanoparticles on PMMA's characteristics. The impact of typical organic (DSNP) and inorganic (TiO_2_NP) nanoparticles on the mechanical characteristics of PMMA was not thoroughly investigated. One of the objectives of this work is to enhance the mechanical characteristics of PMMA used in dentistry by using various nanoparticles. Choosing the best option for improving mechanical qualities appears crucial; thus, the question of material selection must be studied.

Preparing low-cost organic nanostructures such as DSNP as reinforcing materials for PMMA for denture base applications is the uniqueness and significance of this study. In addition, these organic nanoparticles have the potential to enhance the mechanical and tribological characteristics of PMMA composites. This research aims to analyze and compare the mechanical and tribological characteristics of PMMA reinforced with DSNP and TiO_2_NP for applications involving denture bases. Focusing on using nano-organic filler materials minimizes the production cost of denture base composites.

## Materials and methods

### Base material

The benchmark material was handed with a nanocomposite in this experiment. Poly (methyl methacrylate) PMMA was used as the composite matrix, and Acrostone (Cairo-Egypt supplies) was the PMMA matrix material. The mechanical and physical properties of the PMMA material are illustrated in Table 1^32^. Date seed nanoparticle DSNP and titanium dioxide nanoparticle TiO_2_NP are used as reinforced materials. The mechanical and physical properties of the TiO_2_NP material are illustrated in Table 2^32^.TiO_2_NP was supplied by (Morgan Company, Cairo, Egypt), with an average size of ∼ 30 nm.

**Table 1:** The physical and mechanical properties of neat PMMA.

Property	Time of solubility	Setting time	Brinell hardness	Bending strength	Absorbability	Solubility	Resistance to impact
Value (max or min)	4	7	120 Mpa	65.5 Mpa	Max. 32 mg/m	Max. 8 mg/mm 3	Min. 0.40 J/cm 2

**Table 2:** The physical and mechanical properties of neat TiO_2_NPs.

Property	CAS	Purity	APS	Molecular weight	Color	Density	Melting point
Value	13463-67-7	99.9%	30 nm	79.866 g/mol	White	4.23 g/cm^3^	1843 °C

**Figure 1:**
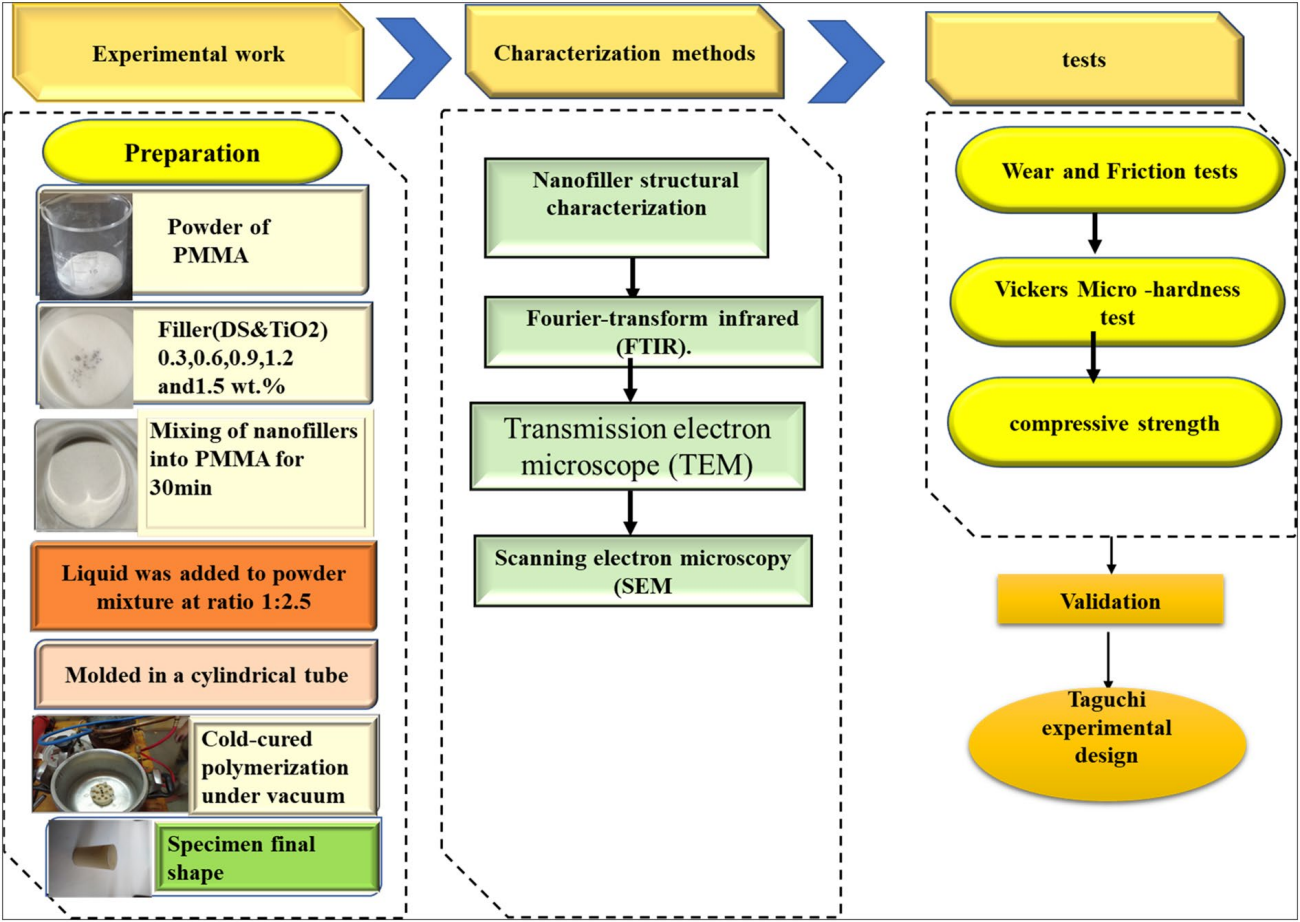
An overview schematic of the methodology present in the work.

The DSNP materials were obtained from the Material Research Lab in this investigation. First, the seeds were air dried before being ground for 24 h at 70 °C. An X-ray diffractometer measured an average particle size of ∼ 20 nm after the seeds were ground in a ball mill for 140 h to achieve the necessary nanoscale particle size. The physical properties DSNP are illustrated in Table 3. Figure 1 illustrates an overview schematic of the methodology present in the work.

**Table 3:** The physical properties of DSNP .

Property	Purity	APS	Color	Density
Value	90%	20 nm	Brown	1.3 g/cm^3^

### Nanofiller structural characterization

DSNP and TiO_2_NP were made with Cu-K radiation at 15 m, a scanning angle of 2°, an ampere current of 25 m A, a speed of 4 s, and a voltage of 40 kV to figure out the crystal structure of the powder. The Scherrer equation^35^ was used to calculate the average size of a crystallite (or grain). The diffraction patterns of DSNP and TiO_2_ NP powders were compared to those in the literature^36,37^, and a transmission electron microscope (TEM; model JEOL JEM-2010) was used to determine nanoparticle size. The average diameter of produced nanoparticles was 22 and 30 nm, respectively.

### Preparation specimens

PMMA/TiO_2_NP and PMMA/DSNP nanocomposites were fabricated by the self-curing method. Specimens were prepared at constant loadings of 0.0, 0.3, 0.6, 0.9, 1.0, 1.2, and 1.5 wt%. Then, hardener liquid (PMMA monomer) was added to the powder mixture and mixed at a ratio of 1:2. To obtain a homogenous mixture; these materials were mixed for 20 min. Afterward, the prepared nanocomposites were poured into a cylindrical tube for 2 h at room temperature and 1.4 bar to obtain the final shape. Hence, the nanocomposites were molded in a cylindrical mold and kept at room temperature (35 ± 2 °C) for 2 h to get the final shape of the samples. The manufactured samples of PMMA (TiO_2_NP and DSNP) have 8 mm diameter and 30 mm length and were cut into suitable sizes for further tests^38^.

## Characterization of PMMA/DSNP and PMMA/TiO_2_NP

PMMA/DSNP and PMMA/TiO_2_NP composites were characterized using mechanical tests, tribological tests, microstructure and worn surfaces examination (SEM), and Fourier-transform infrared spectroscopy (FTIR).

### Mechanical tests

#### Vickers microhardness test

(EVERYONE MH6) following ASTM E 92. The measurements were made using a pyramidal diamond indenter with a facing angle of 136°, 10–100 indentation load, and a load dwell time of 12 s. VHN was calculated as follows:

1 $$VHN=1854.4 \left(\frac{P}{{d}^{2}} \right)$$

where: P is the applied load, gf, and d is the mean diagonal length of the indentation, μm. To get accurate test results, each specimen surface was tested by five indentations from the center to the external periphery of the sample, and average values were reported.

#### Compressive strength

PMMA nanocomposites were put through their paces using a variety of filler reinforcements, all according to the ASTM D695 standard. The tests were done on computerized universal testing equipment (UH series, Shimadzu Corporation) with a crosshead speed of 6 s. They were done at room temperature.

### Tribological tests

#### Wear test

The Pin-on Disk (Wear & Friction Monitor TR-20) following ASTM G 99-95a. The pin was held against the bottom of a 60 mm-diameter wear track on a rotating disc. Dead weights were used to push the pin against the disc. The samples were put through a wear test with different normal loads and a sliding speed of 2 to 4 m/s.

#### Coefficient of friction test

Coefficient of friction (Cof) µ of the samples was measured during the wear test at the same conditions. µ was expressed by dividing the friction force by the applied load. Friction force was recorded each millisecond by a calibrated data logger connected with a load cell of 40 kg. Also, the averaged value of µ from three tests of each specimen was reported.

### Microstructure and worn surfaces examination

The microstructure and worn surfaces of the present samples were examined using SEM. The microstructure was examined to ensure uniform distribution of the NP content within the PMMA matrix material. Also, after the wear and friction tests were completed, worn surfaces were examined to study the effect of adding NP content on improving the tribological properties of the PMMA material and studying the occurring wear mechanism.

### FTIR characterization

FTIR spectrophotometer was recorded using a Bruker (Vertex 70 FTIR-Raman) spectrophotometer. It was done by scanning the pure PMMA, PMMA/1.2 wt% TiO_2_NP, and PMMA/1.2 wt% DSNP. Infrared light was employed to scan the molecular structure of the test samples. Also, the utility of infrared spectroscopy arises due to different chemical structures that produce different spectra.

## Results and discussion

### Microstructures of DSNP and TiO_2_NP samples

**Figure 2:**
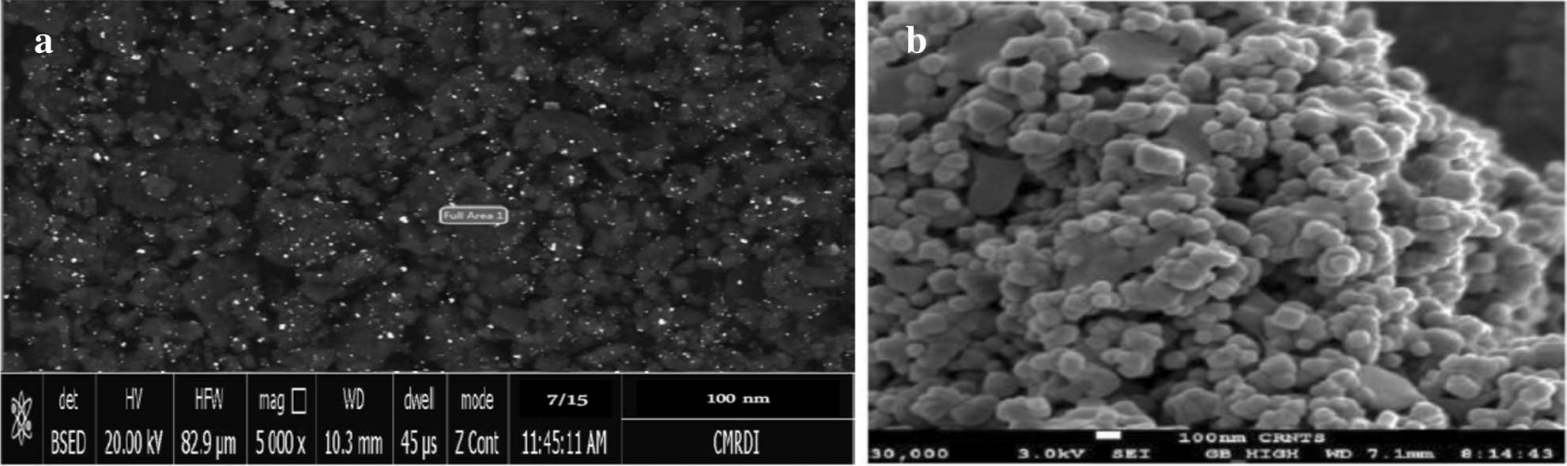
Sample SEM-based morphology images: (**a**) DSPN, and (**b**) TiO_2_NP.

The DSNP and TiO_2_NP microstructures were explored using a scanning electron microscope (SEM). Figure 2 depicts a sample SEM image for each material. Although the form and scale of DSNP and TiO_2_NP particles are highly variable in these images, each particle appears essentially spherical. Similar observations about nanoparticle shapes have been previously reported^35^. These shape and size variations enhance the reinforced materials' mechanical properties.

**Figure 3:**
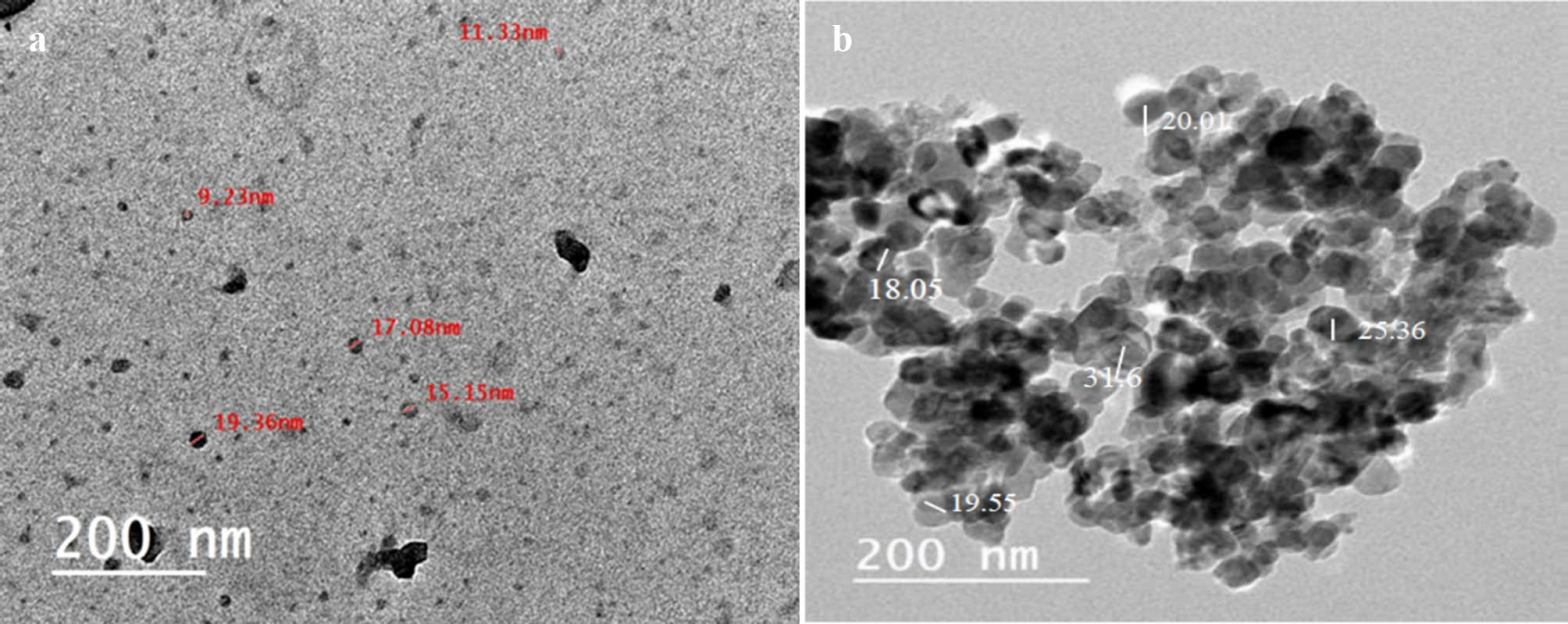
TEM of (**a**) prepared DSNP, (**b**) as received TiO_2_NP.

The morphology and particle size of the ball DSNP powder obtained from TEM are presented in Fig. 3a. The results demonstrate that the DSNP particles were successfully converted into nano-sized particles via ball-mill processing, and an approximate diameter of ≈ 20 nm. On the other hand, Fig. 3b shows the TiO_2_NP morphology and particle size. Indeed, the TiO_2_NP particles have a spherical shape, a clumped distribution, and an approximate diameter of 20 to 30 nm. All these TEM results are in good agreement with the SEM ones.

### Effect of the filler on the mechanical characteristics

#### Effect of the filler type and loading on the wear rate

The material characteristics and operating conditions generally influence material wear. Numerous physical, mechanical, and tribological properties of polymers can be enhanced by supplementing these polymers with particles and fillers of different types and loading conditions. Fillers have been recently treated with organic or inorganic materials to improve filler dispersion in polymer composites. Dispersion and interfacial strength between the filler and matrix materials have also been improved in several research studies^35,39^ using organic or inorganic fillers.

**Figure 4:**
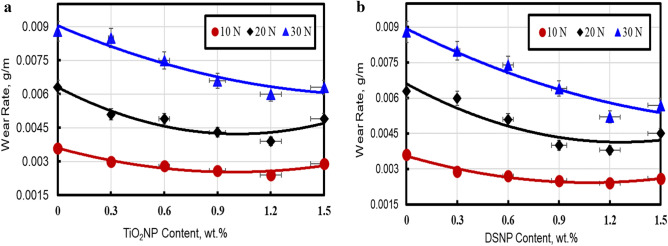
Wear rate as a function of the filler weight and load for the tested nanocomposites: (**a**) PMMA/TiO_2_ NP, and (**b**) PMMA/DSNP.

Figure 4a and b depicts the relationship between the filler weight (wt%) and the wear rate (g/m), for a normal load (L) ranging from 10 to 30 N and an abrading distance (D) of 450 m. Obviously, the wear rate decreased as the filler wt% was increased from 0 to 1.2%. This decrease in wear loss is due to the large nanoparticle surface area, which leads to a large interfacial area between the polymer and the nano-filler materials. This enlarged polymer-filler interface improves the mechanical properties of both the PMMA/DSNP and PMMA/TiO_2_NP nanocomposites. Wear resistance is also improved since the nanoparticles transmit friction, boosting the nanocomposite load-carrying capacity.

Moreover, the results show that the wear resistance was improved by 22%, 57.62, and 68.67% by adding 0.3, 0.9, and 1.2 DSNP wt%, respectively. These results agree with those reported by Elkhouly et al.^29,36,37^, who found that good resistance wear can be obtained for polymers with date-seed nano-powder of 30 nm in nanoparticle diameter. In addition, the results demonstrate that the wear resistance was improved to 19.5%, 55.62, and 61.66% by adding 0.3, 0.9, and 1.2 TiO_2_NP wt%, respectively. These results are in concordance with the conclusions of Nafea et al*.*^40^ who reported a 58.33% reduction in wear rate for the nanoparticle-filled PMMA composites compared to the unfilled PMMA polymers.

On the other hand, the wear rate increased as the normal load was raised from 10 to 30 N. This can be ascribed to the cutting edges becoming covered in wear detritus with increasing load. Furthermore, the nanocomposites under examination show an increase in the wear rate when the typical load increases. For the wear rate to increase, there must be an increase in the load applied to the matrix surface. Related results were also reported earlier^32,36,37^.

For the PMMA matrix, the results in Fig. 4 show also that the wear resistance is strengthened more by the DSNP fillers compared to the TiO_2_NP ones. This relative strength of the DSNP-filled composites is due to the DSNP nanoparticle distribution in the PMMA nanocomposite, where the PMMA matrix is more isolated from abrasive particles, and the adhesion between molecules is stronger compared to TiO_2_NP-filled composites. Specifically, the DSNP nanoparticles were well-infused into the PMMA matrix and hence increased wear resistance by 2.9% compared to the TiO_2_NP ones. Similar results were also reported earlier^17,36–39^.

Based on these results, the recommended composite parameters are a 1.2 wt% with the DSNP filler at a normal load of 10 N. The results also agree with Ramesh^41^ who examined composites of organic date-seed particles and epoxy polymers.

Furthermore, Fig. 4a and b show increasing wear trends at 1.5 wt% of DSNP and TiO_2_NP fillers at higher loading levels (due to the reduced interaction and de-bonding between the nanoparticles and the PMMA matrix). Hence, at a higher nanoparticle loading, the interfacial adhesion between the particles and matrix is insufficient to resist the sliding forces, resulting in higher wear rates^40–42^.

#### Effect of the filler type and loading on the coefficient of friction

**Figure 5:**
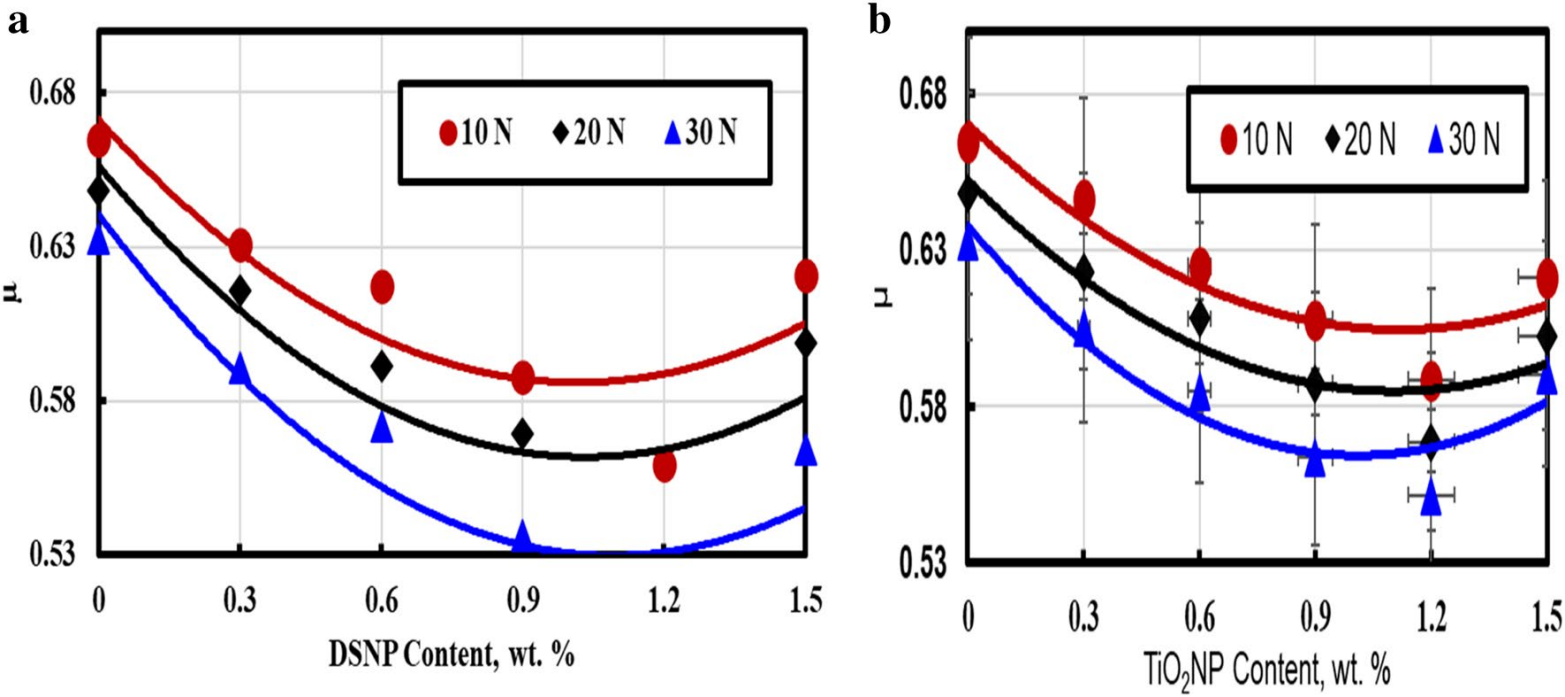
Coefficient of friction as a function of the filler weight and load for the tested nanocomposites: (**a**) PMMA/DSNP, and (**b**) PMMA/TiO_2_NP.

Figure 5a and b illustrate the relationship between the variations in the filler weight (wt%) and the coefficient of friction (COF) µ. The obtained values of µ were checked against the DSNP and TiO_2_NP nanofiller contents at different loading levels (10 to 30 N). In comparison to the µ values for pure PMMA polymers, a considerable improvement was achieved when nanoparticles were added. This agrees well with earlier results^40,41^.

Notably, Fig. 5 shows that a nanofiller can increase the friction resistance. In particular, that friction resistance was improved to 50.19%, 85.18%, and 88.54% by adding 0.3, 0.9, and 1.2 DSNP wt%, respectively. Also, the results show that the friction resistance was improved to 48.13%, 83.18% and 87.18% by adding 0.3, 0.9, and 1.2 TiO_2_NP wt%, respectively. These results can be justified by the reduced shear strength of the PMMA matrix, because of the temperature buildup between the sliding disc and the contact zone of the specimen^42,43^. The results also agree with the conclusions of Hassan et al*.*^41^ who combined Al_2_O_3_ NP fillers with PMMA polymers and showed a reduction from µ = 25.47 by 24.28% under different loading levels.

Based on the results shown in Fig. 5, the best parameter settings are 1.2 wt% for the DSNP filler at a normal load of 10 N. These settings are in good agreement with earlier reported results^39,42,44^. On the other hand, friction resistance decreased with the addition of the DSNP and TiO_2_NP fillers at up to 1.5 wt%. The is due to the weak interaction between the PMMA matrix and the nanoparticles at higher concentrations, agglomeration, and porosity in the samples^43^.

**Figure 6:**
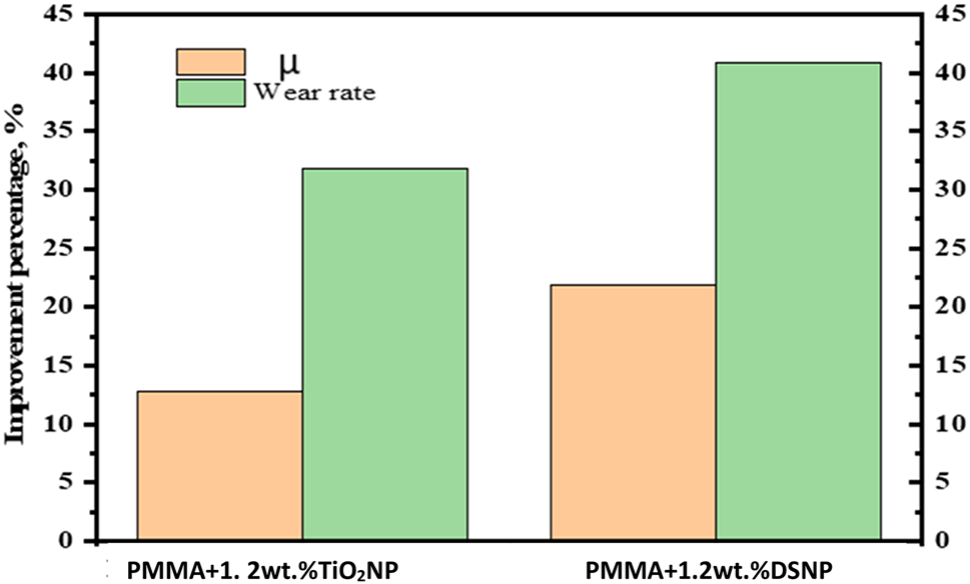
Improvement percentages of WR and µ at 1.2 wt% NP under an applied load of 30 N.

WR and COF (µ) result notably recorded the highest improvement percentage at 1.2 wt% DS NPs by 13.33 and 10.38%, respectively, compared to 1.2 wt% TiO_2_ NP as shown in Fig. 6. This strength of the DS NPs-filled composites is due to the DSNP distribution in the PMMA nanocomposite, where the PMMA matrix is more isolated from abrasive particles, and the adhesion between molecules was stronger compared to TiO_2_NP-filled composites. Specifically, the DSNP have an average size of ∼ 20 nm, which was smaller than that of TiO_2_ + NP, which have an average size of ∼ 30 nm. Therefore, DSNP were well-infused into the PMMA matrix; hence, they increased wear resistance and µ compared to TiO_2_ NP.

#### Effect of the filler weight and type on hardness

**Figure 7:**
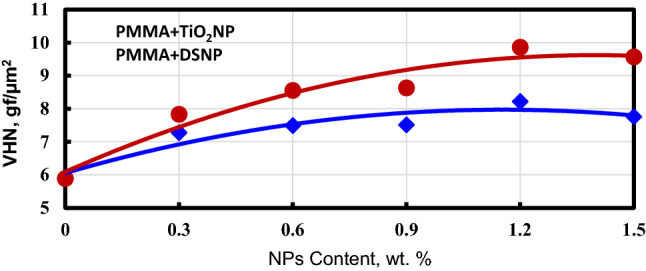
Micro-hardness numbers are functions of the examined nanocomposites' filler weight.

Measurements of the Vickers hardness number (VHN) of the examined PMMA-nanofiller composites are depicted in Fig. 7. With increasing the filler concentration, the hardness increased. Furthermore, adding nanofillers to the PMMA matrix (with 1.2 wt%) raised the hardness by 30.76% compared to the filler-free PMMA materials. This result is in good agreement with that of El-Tamimi et al.^45^ who reported an 11% hardness improvement due to the introduction of nanofillers.

The abovementioned hardness improvement is due to the strong interfacial adhesion of the nanofillers with the PMMA matrix. Indeed, nanofillers have been used as reinforcing elements in polymer-matrix nanocomposites, to enhance their load-bearing capacity^40,45^. Figure 7 shows that the PMMA-DSNP hardness is 5.35% higher than that of the PMMA-TiO_2_NP composite. The reason is that the date-seed nanoparticles function as natural lubricants. This finding is in good agreement with the results reported in^46^.

#### Effect of the filler type and loading on the compressive strength

**Figure 8:**
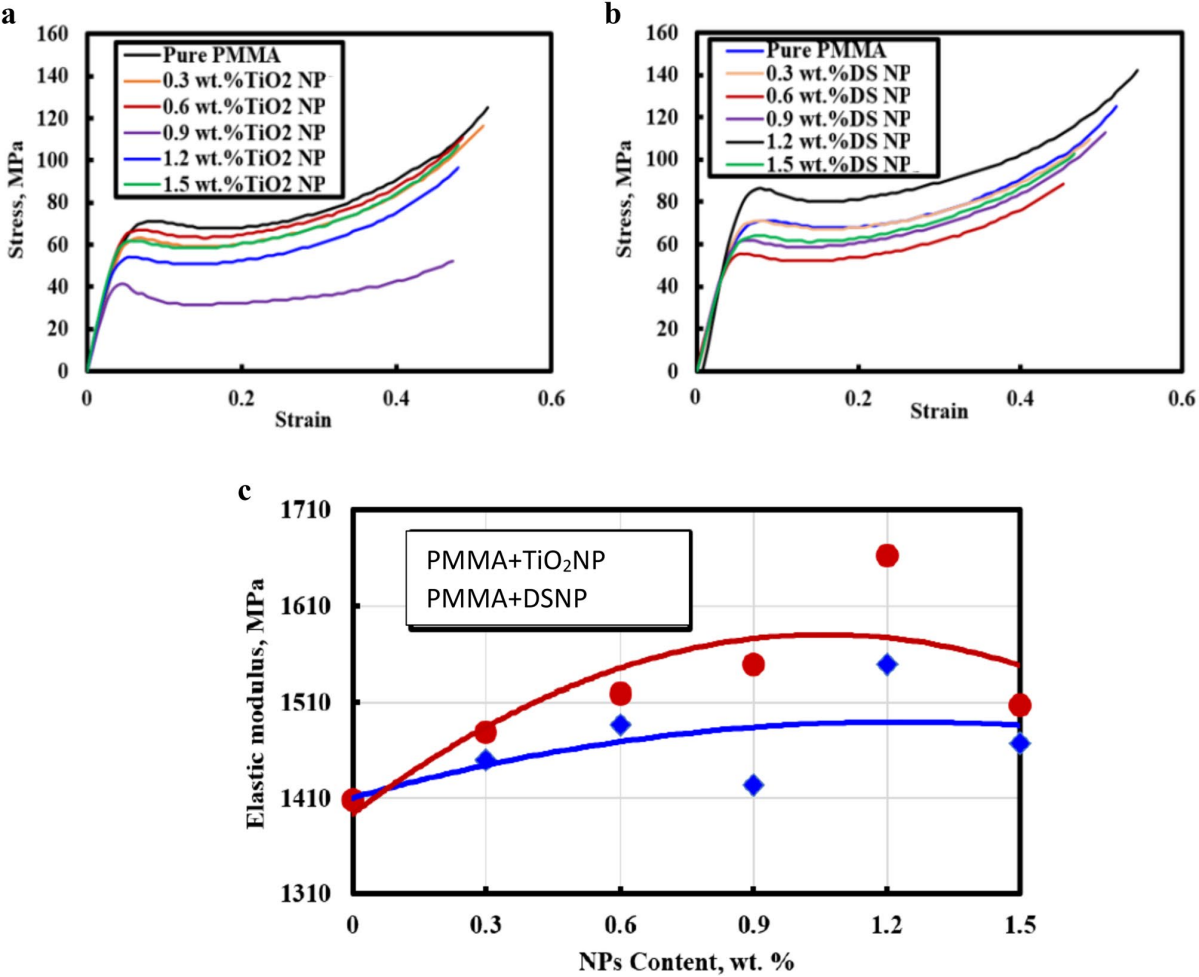
Stress–strain curves for the examined nanocomposites. (**a**) PMMA-TiO_2_NP, (**b**) PMMA-DSNP, (**c**) Modulus of elasticity for the examined nanocomposites.

Compressive strength tests were carried out on raw PMMA polymer specimens and their composites with varied percentages of the DSNP and TiO_2_NP fillers, as shown in Fig. 8a and b. Stress–strain curves were thus constructed to estimate the moduli of elasticity of the examined composites. The results show that the compressive strength was improved to 145 MPa and 120 MPa by adding 1.2 wt% of DSNP and TiO_2_NP fillers, respectively. In fact, with nanofiller addition, the moduli of elasticity of the PMMA composites showed a gradually increasing trend, followed by a progressively decreasing trend as the amount of the nanoparticles increased. Moreover, the total saturation of the examined composites may be justified by the fact that the normal load was transferred through the interfacial surfaces to the strongest material, namely, the nanoparticles^47,48^. Our results agree with those of Shirkavand et al.^46^, who found that the PMMA compressive strength increased as the resin strength increased with increasing filler weight. As illustrated in Fig. 8, the poor interaction between the PMMA matrix and the DSNP or TiO_2_ NP fillers at 1.5 wt% leads to less effective stress transfer between the PMMA polymer and the strengthening materials. As initially demonstrated, this result is in good agreement with those reported in^4,41,42^. Figure 8c shows that the modulus of elasticity rises with increasing nanofiller concentration, as expected by Chatterjee et al.^49^, following the law of mixtures. To transfer the applied stress from the PMMA matrix to the nanofillers, the moduli of elasticity of the DSNP and TiO_2_NP nanofillers must be high. In summary, a detailed comparison of the mechanical properties of the PMMA-DSNP and PMMA-TiO_2_NP nanocomposites with the properties of other nanofiller-based composites are shown in Table 4.

**Table 4:** Comparison of the mechanical properties of the PMMA-DSNP and PMMA-TiO_2_NP nanocomposites with those of other nanofiller-based composites.

Material	Wear resistance %	Coefficient friction resistance %	Hardness %	Compressive strength (MPa)	Ref.
PMMA-DSNP	66.67	88.54	40	145 MPa	Present work
PMMA-TiO_2_NP	61.66	87.18	35	120 MPa	Present work
PMMA-Al_2_O_3_NP	59.3	25.47	14	110 MPa	^40^
PMMA-ZrO_2_NP	58.33-	69.31–64.25%	11	115 MPa	^32,47^
PMMA-ZrO_2_NP	55.5%	–	11	–	^45^
DSNP-MDPE	45%	–	35	–	^39^
DSNP-PET	70%	–	40	–	^35^
DSNP-epoxy	71%	–	–	–	^27^
DSNP-vinyl ester				95 MPa	^38^

### Spectral analysis using FTIR

**Figure 9:**
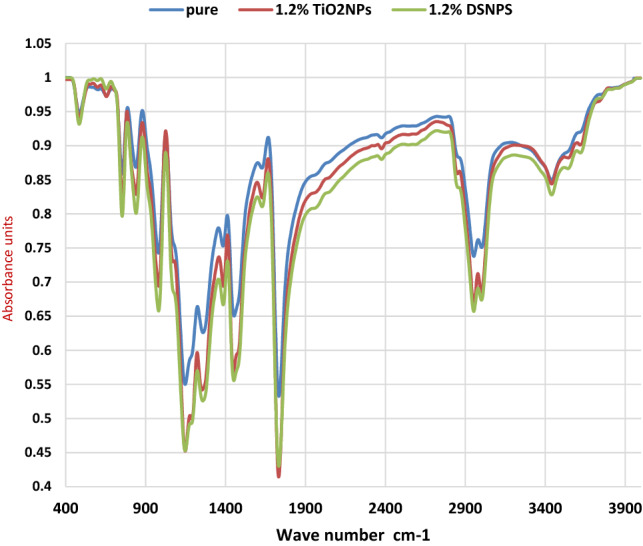
FTIR spectral curves for the raw and composite polymers.

The spectral transmittance curves of the raw PMMA polymer (with no nanofillers) and their nanocomposite counterparts with 1.2% of the DSNP and TiO_2_NP fillers have been constructed using FTIR spectral analysis, as shown in Fig. 9.

The FTIR technique shows the IR spectra of the free PMMA specimens. For the free PMMA polymer in Fig. 9, the peaks at 1552, 1139, 885, and 781 cm^−1^ represent C=C–H stretching vibrations, C–O–C stretching in ethers, C–O–C stretching in oxirane, and CH_2_ rocking modes, respectively. The chemical structure of the PMMA-TiO_2_NP composite has been identified using ATR-FTIR. The strong bands within the wavenumber range of 400–700 cm^−1^ confirm that the Ti–O–Ti network structures are associated with the C=C weak stretching vibrations and the C–H stretching vibrations of CH_3_.

Furthermore, two strong bands appear at 2375 cm^−1^ and 2953 cm^−1^ because of the –CH2 stretching modes of the PMMA matrix. These two peaks are flattened with the nanofiller addition. In particular, peak flattening and shifting happened because of the (C–O) and (C=O) bonds with the TiO_2_NP fillers. This result is in good agreement with the those reported in^32^.

For the PMMA-DSNP composite (with 1.2 wt%), the strong bands in Fig. 9 at the wavenumbers of 4450 cm^−1^ and 550 cm^−1^ are due to the C=C weak stretching vibrations and the C–H stretching vibrations of CH_3_, respectively. The strong bands at 1100–1600 cm^−1^ are due to the C = C weak stretching vibration and C–H stretching vibration of CH_3_, respectively. In Fig. 9, the bands at the wavenumbers of 3470 and 2942 cm^−1^ can be attributed to CH_2_ splitting^27,34,47^. The drop of these peaks reflects the physicochemical interaction of the PMMA polymer with the nanoparticles at the contact surfaces when the composites were sintered.

### Worn surface test

**Figure 10:**
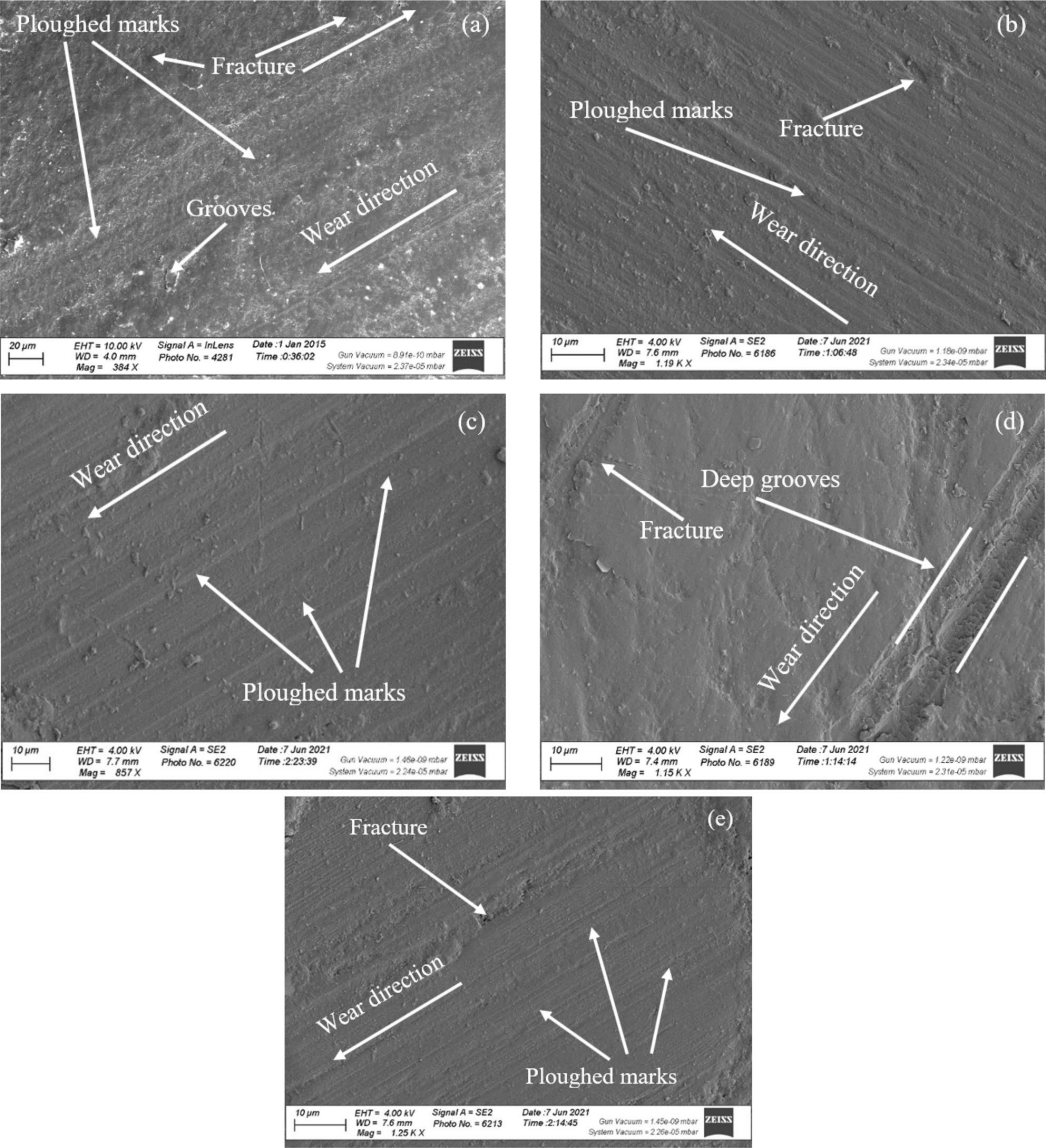
SEM micrographs of the worn surfaces for the raw and reinforced polymers. (**a**) pure PMMA, (**b**) PMMA-DSNP (1.2 wt%), (**c**) PMMA-TiO_2_NP (1.2 wt%), (**d**) PMMA-DSNP (1.5 wt%), and (**e**) PMMA-TiO_2_NP (1.5 wt%).

A scanning electron microscope (SEM) was used to inspect the worn surfaces of raw PMMA samples and PMMA samples reinforced with DSNP and TiO_2_NP fillers (with 1.2 and 1.5 wt% for each filler type). Figure 10a depicts a continuous groove and a deeper groove on the surface parallel to the sliding path. Some more refined PMMA grains were scratched off during the wear process. Figure 10b–d show the worn surfaces of the PMMA material combined with DSNP and TiO_2_NP fillers (with fractions of 1.2 and 1.5 wt% for each filler type). Compared to the raw PMMA surface, the worn surfaces of the nanocomposites had shallower and narrower furrows. The importance of the interfacial bonding between the PMMA and DSNP materials is thus clearly established. Indeed, the DSNP fillers act as lubricant substances at contact surfaces. Moreover, the samples showed no porosity defects, indicating the effectiveness of the production process in creating sound acrylic nanocomposites at different percentages.

Additionally, more ploughed marks and porosity configurations were observed on worn surfaces when DSNP and TiO_2_NP fillers were added to the PMMA matrix with a 1.5 wt%, as shown in Fig. 10c and e. These patterns are due to the porous arrangement of the weak nanofiller interaction with the PMMA material at high concentrations. These results agree well with those reported in^17,32,33,42^.

### Economical comparison between the DSNP and TiO_2_NP fillers

Material expenses account for around 40% of commodity costs. So, reducing material costs would increase profitability. Indeed, DSNP fillers are typically considered waste products with no utility, whereas TiO_2_NP fillers are generally expensive. As a result, we substitute TiO_2_NP fillers with low weights for the DSNP fillers. When PMMA nanocomposites were strengthened with 1.2% of DSNP and TiO_2_NP fillers, the wear resistance increased by 41% and 31%, respectively. Also, the TiO_2_NP fillers cost 60$/kg, whereas the DSNP powder costs 4$/kg (1$/kg cost of DSNP material + 3$ cost of DSNP nanomaterial). With a filler weight of 1.2%, it has been shown that the PMMA-TiO_2_NP and PMMA-DSNP costs are $76/unit and $14/unit, respectively. At the same time, the cost of the raw PMMA polymer is $10/unit. Despite the cheaper raw polymer, the DSNP fillers still have the advantage of improving the mechanical qualities of the PMMA nanocomposites^34,40^.

## Validation

### Taguchi experimental design

**Figure 11:**
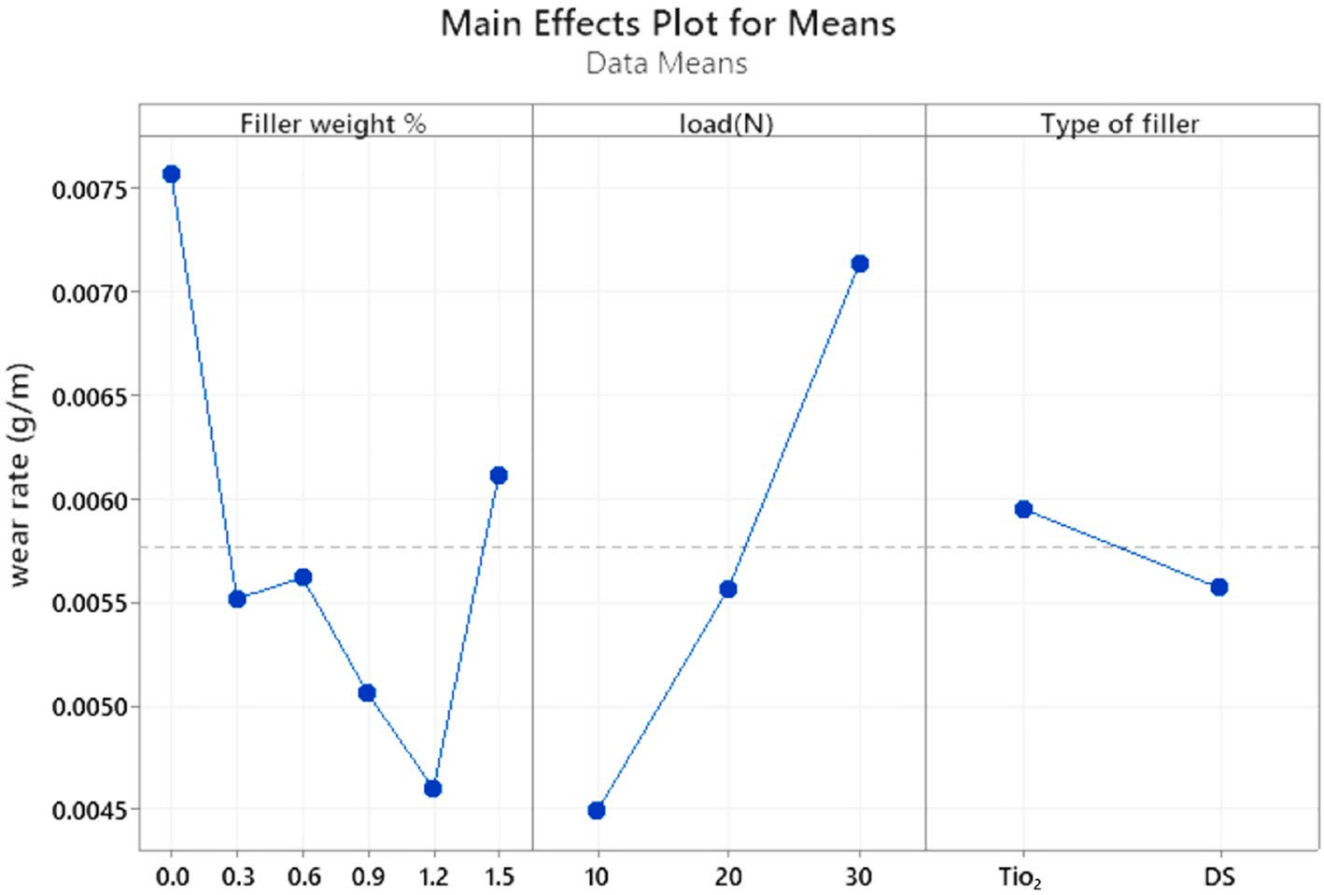
Main effects plot for mean wear rate.

The Taguchi method employs an orthogonal array of arrays to investigate the effects of experimentally manipulated variables. The orthogonal array was designed using the Taguchi approach using three factors: one component with five levels, another with three levels, and the third with two levels. The filler weight percent was 0–1.5%, the normal load (N) was 10 to 30 N, and the type of filler was (DSNP and TiO_2_NP). The experiments were based on the L36 array. The analysis was done on MINITAB 20. Figure 11 shows how the nanocomposite (WR) and the filler weight percent, the filler types, and the normal load (L) are linked together to make sense^27,28,41^.

As filler weight increases, we see a decrease in WR. This study shows how filler weight, normal load (N), and type of filler all affect the nanocomposite water resistance (WR). When analyzing these parameters, it became clear that the filler weight % was the most important, closely followed by the type of material and the normal load.

### Confirmation test

Section “Effect of the filler type and loading on the wear rate” discusses results remarkably close to the Taguchi confirmation trials, specifically 1.2 wt% of filler and a normal load of 10 N, which are suggested and ideal values. The experiments and the theory appeared in complete agreement, at least on the surface. DSNP filler is ranked first before TiO_2_NP, closely matching the results of the Taguchi confirmation trials, which show that DSNP filler is better than TiO_2_NP filler^28,50^. Discusses results remarkably close to the Taguchi confirmation trials, specifically 1.2 wt % of filler and a normal load of 10 N, which are suggested and ideal values. The experiments and the theory appeared in complete agreement, at least on the surface. DSNP filler is ranked first before TiO_2_NP, closely matching the results of the Taguchi confirmation trials, which show that DSNP filler is better than TiO_2_NP filler^28,50^.

## Conclusion

Poly (methyl methacrylate) (PMMA) is used widely in dental applications, but its mechanical properties are low. This research fabricated PMMA matrix material reinforced with two nanofillers of DSNP and TiO_2_NP. Also, the mechanical and tribological behavior of the present nanocomposites were investigated for denture base applications. Mechanical properties, including elastic modulus and microhardness, and tribological properties, including WR and µ under a normal load at 30 N of pure PMMA were improved with increasing DSNP and TiO_2_NP at 1.2 wt%, respectively. The results indicated that WR and µ resistance of PMMA was improved by 88.54% and 66.67% by adding 1.2 wt% DSNP, respectively.

Composites of PMMA/DSNP recorded higher mechanical and tribological properties than that of PMMA/TiO_2_NP composites at 1.2 wt% nanofiller content. In the meantime, worn surfaces of the composites after the wear test were enhanced at 1.2 wt% nanofiller content. the nanofiller concentration up to 1.5 wt% led to agglomeration, porosity formation, and decreasing mechanical and tribological properties of nanocomposites. Additionally, worn surfaces showed more ploughed marks and porosity at 1.2 wt% of (DSNP and TiO_2_NP). Within the confines of this study, it can be concluded that the optimum nanofiller type and its concentration are DSNP at 1.2 wt% for improving all mechanical and tribological properties of pure PMMA.

## Data Availability

The datasets used and analyzed during the current study are available from the corresponding author on reasonable request.

## Abbreviations

PMMA: Poly (methyl methacrylate)
DSNP: Date seeds nanoparticle
TiO_2_NP: Titanium oxides nanoparticle
SEM: Scanning electron microscope
µ: Coefficients of friction
WR: Wear rate
MDPE: Medium-density polyethylene
PET: Polyethylene terephthalate
FTIR: Fourier-transform infrared
D: Abrading distance
L: Normal load
PEO: Poly (ethylene oxide)
